# A novel *F8* variant in a Chinese hemophilia A family and involvement of X-chromosome inactivation: A case report

**DOI:** 10.1097/MD.0000000000033665

**Published:** 2023-05-05

**Authors:** Honghong Zhang, Yinjie Li, Xiaojuan Lv, Yuchan Mao, Yixi Sun, Ting Xu

**Affiliations:** a Department of Pediatrics, Hangzhou Children’s Hospital, Hangzhou, Zhejiang, China; b Key Laboratory of Reproductive Genetics, Ministry of Education (Zhejiang University), Hangzhou, Zhejiang, China; c Department of Reproductive Genetics, Women’s Hospital, School of Medicine, Zhejiang University, Hangzhou, Zhejiang, China.

**Keywords:** androgen receptor gene (*AR*) assay, factor VIII gene, hemophilia A, X-chromosome inactivation (XCI)

## Abstract

Hemophilia A (HA) is an X-linked recessive bleeding disorder, which shows factor VIII (FVIII) deficiency caused by genetic variant in *F8* gene.

**Patient concerns:**

Males with *F8* variants are affected, whereas female carriers with a wide range of FVIII levels are usually asymptomatic, it is possible that different X-chromosome inactivation (XCI) may effect the FVIII activity.

**Diagnoses:**

We identified a novel variant *F8*: c.6193T > G in a Chinese HA proband, it was inherited from the mother and grandmother with different FVIII levels.

**Interventions:**

We performed Androgen receptor gene (AR) assays and RT-PCR.

**Outcomes:**

AR assays revealed that the X chromosome with the *F8* variant was severely skewed inactivated in the grandmother with higher FVIII levels, but not in the mother with lower FVIII levels. Further, RT-PCR of mRNA confirmed that only the wild allele of *F8* was expressed in the grandmother, with lower expression in the wild allele of the mother.

**Lessons:**

Our findings suggest that *F8*: c.6193T > G could be the cause of HA and that XCI affected the FVIII plasma levels in female carriers.

## 1. Introduction

Hemophilia A (HA) is a bleeding disorder caused by a partial or total deficiency in the coagulation factor VIII (FVIII), which results in prolonged oozing after injuries, tooth extractions, or surgery, as well as delayed or recurrent bleeding prior to complete wound healing.^[1,2]^ Based on the coagulation FVIII activity (FVIII:C), HA is classified into 3 phenotypes: severe (FVIII:C < 1%), moderate (1%–5%), and mild (5%–40%).^[3]^

FVIII coding gene *F8*, which spans approximately 186 kilobases (kb) of genomic DNA and has 26 exons, is located at the distal end of the long arm of the X chromosome (Xq28).^[1,4]^ According to the Human Gene Mutation Database Professional 2021.2 database and *F8* variant database (http://www.factorviii-db.org/index.php), more than 3000 variants of *F8* have been reported. The *F8* gene is translated into a 2315 amino acid polypeptide with 6 structural domains: A1-A2-B-A3-C1-C2.^[5]^ Inversions in intron 22 and intron 1 are the most common gene defects in severe HA patients, accounting for 45% to 50% and 0.5% to 5% of cases, respectively.^[6,7]^ Other different *F8* variants cause the remaining severe, moderate, and mild patients.

As an X-linked recessive disorder, males with *F8* variants are affected. In general, heterozygous females are asymptomatic carriers of the disease, the median FVIII:C level of carriers was 60% compared with 102% in noncarriers, however, approximately 30% of heterozygous females have clotting activity below 40% and are at risk for bleeding, even if some females are affected.^[8–10]^

46, XX female is a mosaic of 2 cell types that expresses both maternal and paternal X chromosomes. Normally, cells have equal inactivation (50:50) of the maternal or paternal X chromosome, but some females have preferential inactivation of 1 X chromosome, which is known as skewed X-chromosome inactivation (XCI).^[11,12]^ In females, skewed XCI can result in phenotypic heterogeneity of many X-linked disorders, disease severities were positive correlation with the activated ratio of mutated X chromosome.^[13–15]^ Recently, some reports revealed that XCI might be a modifier of FVIII plasma levels, leading to the low expression of clotting factor levels and bleeding symptoms in HA carriers.^[16–18]^ Correlation between XCI patterns and FVIII levels in HA female carriers with the same *F8* variant contributed to further confirm this theory. In this study, we reported a novel variant in the *F8* (NM_000132.4): c.6193T > G (p.W2065G) gene in a Chinese HA family. Morever, we discovered that plasma FVIII levels of the female carriers were affected by XCI at the methylated and transcriptional levels using androgen receptor gene (*AR*) assay and RT-PCR.

## 2. Methods

### 2.1. Ethical approval

This study was carried out in accordance with the recommendations of the Ethics Committee of Women Hospital School of Medicine Zhejiang University. Also, in accordance with the Declaration of Helsinki, all participants provided informed consent. Written informed consent was obtained from the parents and other participants of the family for the study. The study protocol was approved by the Review Board of Women Hospital School of Medicine Zhejiang University in China.

### 2.2. Patients

At Hangzhou Children Hospital, a 1-year-old boy was diagnosed with moderate HA (FVIII clotting activity of 2.8%). He had a clinical symptom of prolonged or delayed bleeding or poor wound healing after trauma, such as blood drawing, and his grandmother brother also had similar phenotypes. Prophylaxis with FVIII replacement, the standard of care in HA, was recommended for the patient.

**Figure 1.**
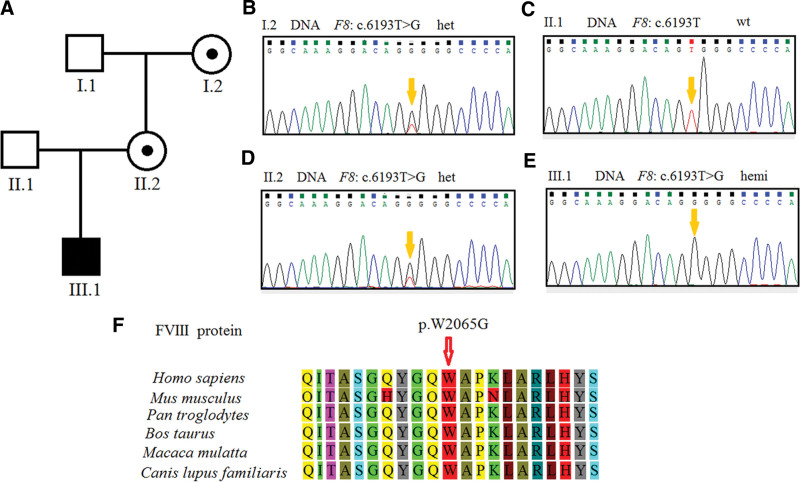
(A) Pedigree of the family. (B–E) Sequence analysis of genomic DNA from family members. The genotypes of *F8* were c. 6193T > G het, c. 6193T wild type, c. 6193T > G het, c. 6193T > G hemi, in I.2 (grandmother) (B), II.1 (father) (C), II.2 (mother) (D), and III.1 (proband) (E). The variant is indicated by yellow arrow. (F) Amino acid alignment of the FVIII protein from several organisms. The position of 2065 residue was highly conserved among different species. FVIII = factor VIII.

His 34-year-old mother and 60-year-old grandmother were carriers with varying FVIII levels (mother: 38.5%; grandmother: 112.1%) (Fig. 1A and Table 1).

**Table 1:** XCI patterns for *AR* markers in blood and FVIII coagulant activity.

	I.2	II.2	III.1
Fragments length	269/286	269/283	269
XCI pattern	92:8	29:71	/
FVIII coagulant activity (%)#	112.1	38.5	2.8

Underlined alleles represent the Xi.

AR = androgen receptor, FVIII = factor VIII, XCI = X-chromosome inactivation.

#Reference: 50–150 (%).

To investigate the genetic cause and prenatal diagnosis for next pregnancy, we performed targeted analysis for intron 22 and intron 1 inversions,^[19]^ next-generation sequencing (NGS) for the proband, and polymerase chain reaction (PCR)-sanger sequence to identify the detected variant. Meanwhile, we used *AR* assays and RT-PCR to determine the XCI status of the 2 females.

### 2.3. DNA/RNA extraction and RT-PCR

We extracted genomic DNA from peripheral blood samples of the patient, his parents and grandmother using the GentraPuregene Kit (Qiagen, Germany), as directed by the manufacturer instructions. Peripheral blood mononuclear cells (PMBCs) were isolated by Ficoll density gradient separation. Total RNA was extracted from PMBCs of the patient, his parents and grandmother using RNAiso Plus (Takara, Japan). Extracted total RNAs were reverse-transcribed using RT Kit (Takara, Japan). Moreover, PCR were performed using GoldStar Best MasterMix (CWBIO, Beijing). DNA/RNA PCR primer sequences are as follows: *F8*-DNA-F:GTGGTACGCGATTGTAGT, *F8*-DNA-R:CATTAAGGCATTCTGTTCTT; *F8*-RNA-F:GCCTTATTGGCGAGCATC, *F8*-RNA-R:ACTTCTGACGGGCACCCT, and sanger sequencing were performed on an ABI 3500 DNA analyzer.

### 2.4. Next-generation sequencing

NGS was performed for the patient by MyGenostics Inc. (Beijing, China). Genomic DNA was fragmented using a S220 Focused-ultrasonicator (Covaris, Massachusetts). For the preparation of standard Illumina libraries, a DNA Sample Prep Reagent Set (MyGenostics, Beijing, China) was used. The amplified DNA was captured with the help of GenCap capture kit (MyGenostics Inc., Beijing, China). The OMIM database was used to obtain the gene panel for hemostasis and thrombotic disease. The biotinylated 100 bp capture probes were designed to tile along the coding exons as well as 50 bp flanking regions of all the genes. The capture experiment was carried out according to the manufacturer protocol. Moreover, the PCR product was purified using SPRI beads (Beckman Coulter) according to the manufacturer protocol. In addition, the enrichment libraries were sequenced for paired-reading of 150 bp on an Illumina HiSeq X ten sequencer.

### 2.5. XCI analysis in peripheral blood

To analyze XCI in terms of DNA methylation, XCI in females was examined using PCR amplification of the *AR* gene, as previously described. Primer sequences are as follows: *AR*-F: TCCAGAATCTGTTCCAGAGCGTGC, labeled by 5`6-FAM (FITC), and *AR*-R: GCTGTGAAGGTTGCTGTTCCTCAT.^[20]^ Degree of skew XCI was calculated using the equation: (d1/u1)/[(d1/u1) + (d2/u2)], d1 and d2 represented the digested alleles of the tested subject, and u1 and u2 represented the undigested alleles.^[21]^

## 3. Results

In order to investigate the possible genetic cause, we performed an intron 22 and intron 1 inversion of the *F8* gene in the proband and found no positive findings (data not shown). Then, the *F8* variant of the proband was detected by NGS. A novel variant of c.6193T > G was discovered in exon 21 of the *F8* gene. This base substitution occurred in the first base in codon 2065 (TGG > GGG), resulting in a missense variant (p.W2065G). By Sanger sequencing, this variant was confirmed in DNA extracted from the peripheral blood in the proband, his parents and grandmother (Fig. 1B–E). His mother II.2 and grandmother I.2 were heterozygous for the variant (Fig. 1B and D), while his father II.1 was of the wild-type genotype (Fig. 1C).

*F8* c.6193T > G variant was not found in the Genome Aggregation Database (gnomAD). In species, tryptophan at position 2065 (p.W2065) was conservative (Fig. 1F). The *F8* c.6193T > G variant was predicted by Variant Taster to be a disease-causing variant. With scores of −11.4 and 0, respectively, PROVEAN and SIFT program analyses of the p.W2065G variant supported the deleterious function of this substitution. With a high score of 1.000, PolyPhen-2 software identified this variant as a potentially harmful variant (sensitivity: 0.00; specificity: 1.00).

**Figure 2.**
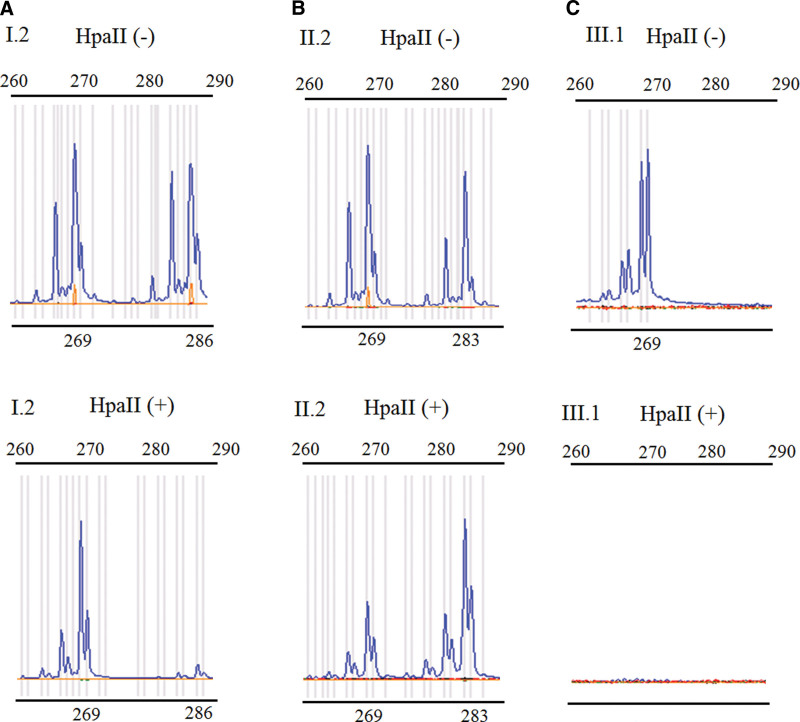
X-chromosome inactivation (XCI) pattern and linkage analyses were based on *AR*. (A) The undigested PCR product of I.2 gave 2 peaks of 269 and 286 bp, while 1 major peak of 269 bp was observed with the HpaII digested product. I.2 exhibited extreme skewing of XCI, and the inactivated X-chromosome was linked with the 269 bp peak of the *AR* PCR products. (B) The undigested PCR product of the proband II.2 gave 2 peaks of 269 and 283 bp. One X-chromosome linked with the 269 bp peak of *AR* was inherited from the grandmother I.2. The product of HpaII digestion also gave 2 peaks, but the 269 bp peak was lower, indicating the X-chromosome inherited from the grandmother I.2 was in preferential expression. (C) The undigested PCR product of III.1 gave 1 269 bp peak, which derived from the X-chromosome of grandmother I.2 and mother II.2, and the *F8* variation also located in the X-chromosome. The digested PCR product of III.1 gave no peak because the X-chromosome of male was active. AR = androgen receptor, PCR = polymerase chain reaction.

Although both the mother and grandmother are carriers, there is a significant difference in coagulant FVIII levels (mother: 38.5%; grandmother: 112.1%). To find out why, we performed XCI pattern analysis using the *AR* gene assay, a PCR-based XCI assay that employs a methylation-sensitive restriction enzyme. As shown in Figure 2, after digestion with the methylation-sensitive restriction enzyme HpaII, only *AR* PCR product on the inactive X-chromosome could synthesize. Furthermore, the origin of the inactivated X-chromosome was determined by segregation analysis. The undigested PCR product of grandmother I.2 yielded 2 peaks of 269 bp and 286 bp, respectively. For the HpaII-digested product, a major peak of 269 bp was observed, which delivered to II.2 and III.1 (Fig. 2). Segregation analysis also revealed that the mutated allele was extremely inactivated (92:8) in grandmother I.2 (Fig. 2A and Table 1). In mother II.2, the inactivated ratio of the mutated allele to the normal allele was 29:71 (Fig. 2B and Table 1), indicating that the mutated allele was preferentially expressed. The undigested PCR product of III.1 gave 1 269 bp peak, which derived from the X-chromosome of grandmother I.2 and mother II.2, and the digested PCR product of III.1 gave no peak because the X-chromosome of male was active (Fig. 2C).

**Figure 3.**
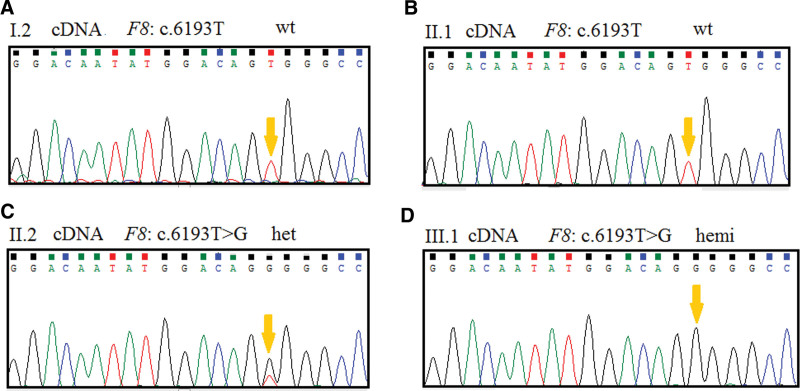
(A–D) Sequence analysis of genomic cDNA from family members. The genotypes of *F8* were c. 6193T wild type, c. 6193T wild type, c. 6193T > G het, c. 6193T > G hemi, in I.2 (grandmother) (A), II.1 (father) (B), II.2 (mother) (C), and III.1 (proband) (D). The variant is indicated by yellow arrow.

RT-PCR of mRNA from PMBCs confirmed that only the wild allele of *F8* was expressed in the grandmother I.2 with the heterozygous variant (Fig. 3A), but the wild allele of the mother II.2 was expressed at a lower level (Fig. 3C). RT-PCR of mRNA from PMBCs of II.1 and III.1 were wild type and hemizygosity, respectively (Fig. 3B and D).

## 4. Discussion

Since the *F8* was first cloned in 1984, an increasing number of *F8* variants have been reported. Here we reported a novel variant c.6193T > G (p.W2065G) of the *F8* gene in a Chinese HA family. The male proband suffered from moderate HA. His mother and grandmother were both asymptomatic carriers with varying FVIII levels. Using *AR* assay and RT-PCR, we confirmed that the active status of the X chromosome with the variant affected FVIII activity.

The *F8:* c. 6193T > G variant results in a single amino acid substitution at codon 2065 (p.W2065G) in the C2 domain of FVIII. Tryptophan is a nonpolar, hydrophobic amino acid, whereas glycine acid, is a polar, neutral amino acid. Another *F8* variant p.W2065R, has been reported at the same location.^[22,23]^

The C2 domain, where the variant is located, is a necessary recognition region for molecules anchoring to procoagulant phospholipid surfaces.^[5,24]^ Via the C2 and A3 domains, FVIII binds to von Willebrand factor to form a stable noncovalent complex that prevents degeneration.^[25,26]^ So, we hypothesized that p.W2065G in the C2 domain of FVIII might affect binding and thus influence FVIII activity, leading to the proband with moderate HA. His mother and grandmother with the same variant, but the FVIII activity were different.

In general, female HA carriers with heterozygous variants are asymptomatic and express about half the normal activity level of the factors, but some have a wide range of clotting factor levels, ranging from the normal range to very low level, with variable bleeding symptoms.^[9,10]^ Many factors involved in the hemophilic females, such as homozygosity or compound heterozygosity for *F8* gene variants, monosomy X, or preferentially skewed inactivation of the normal X chromosome.^[27]^

Estimates of the degree of XCI in many X-linked diseases, such as X-linked mental retardation, *MECP2* duplication syndrome, and Lesch-Nyhan disease, has previously been published.^[28–30]^ While the role of XCI in FVIII levels of female HA groups remains controversial, Orstavik et al firstly reported that absence of correlation between XCI pattern and plasma concentration of FVIII in HA carriers,^[31]^ but recently Garagiola et al found that XCI was significantly skewed in the cohort of hemophilia carriers with low clotting activity, which might contribute to the low expression of clotting factor levels and bleeding symptoms.^[16]^ We thought that the reason for 2 different conclusions might be the interference of other factors, especially the differences of detrimental degrees in different *F8* variants, female carriers with different *F8* variants were not very well suited for exploring the correlation between XCI pattern and FVIII levels. Female carriers in a family with the same *F8* variant contributed to further study. In our study, we reported the mother and grandmother with *F8:* c. 6193T > G, but the FVIII activity of the grandmother was high-normal due to the extreme skewed inactivation on the X chromosome with *F8* variant. Inactivated proportion of the mutated X chromosome in the mother was lower, resulting in the lower FVIII activity.

The reason for extremely skewed XCI in the grandmother was unknown. Some reasons have been proposed in the literature.^[13]^ First, it may only occur by chance. Secondly, it may also be caused by variants in the *XIST* gene responsible for initial inactivation. Thirdly, differential cell growth and/or survival rates may lead to skewed XCI. It has been established that the presence of an X-linked variant results in a growth disadvantage for cells expressing the mutated allele due to selectively mediated favorable skewing. Meanwhile, in cross-sectional studies of populations older than 55 to 60 years old, the skewness of XCI increased significantly with age.^[21,32,33]^ We hypothesized that cells expressing the mutated allele with growth disadvantage were gradually eliminated through long-term selection. Preferential X-inactivation against the *F8* variation increased with age, resulting in high-normal FVIII activity of the grandmother, which still need to be further explored.

In conclusion, we found a novel variant c.6193T > G (p.W2065G) in *F8*, which could cause HA, and FVIII plasma levels in female carriers with the variant might be affected by XCI, which could help with HA genetic counseling and prenatal diagnosis.

## Acknowledgments

We thank all the participants in the present study.

## Author contributions

**Investigation:** Honghong Zhang, Yinjie Li, Xiaojuan Lv, Yuchan Mao, Yixi Sun.

Supervision: Ting Xu.

Validation: Honghong Zhang.

Writing – original draft: Honghong Zhang.

Writing – review & editing: Honghong Zhang.
